# Microglia in action: how aging and injury can change the brain’s guardians

**DOI:** 10.3389/fncel.2015.00054

**Published:** 2015-02-23

**Authors:** Athanasios Lourbopoulos, Ali Ertürk, Farida Hellal

**Affiliations:** ^1^Laboratory of Experimental Stroke Research, Institute for Stroke and Dementia Research (ISD), University of Munich Medical SchoolMunich, Germany; ^2^Laboratory of Acute Brain Injury, Institute for Stroke and Dementia Research (ISD), University of Munich Medical SchoolMunich, Germany

**Keywords:** microglia, stroke, traumatic brain injury, inflammation, aging

## Abstract

Neuroinflammation, the inflammatory response in the central nervous system (CNS), is a major determinant of neuronal function and survival during aging and disease progression. Microglia, as the resident tissue-macrophages of the brain, provide constant support to surrounding neurons in healthy brain. Upon any stress signal (such as trauma, ischemia, inflammation) they are one of the first cells to react. Local and/or peripheral signals determine microglia stress response, which can vary within a continuum of states from beneficial to detrimental for neuronal survival, and can be shaped by aging and previous insults. In this review, we discuss the roles of microglia upon an ischemic or traumatic injury, and give our perspective how aging may contribute to microglia behavior in the injured brain. We speculate that a deeper understanding of specific microglia identities will pave the way to develop more potent therapeutics to treat the diseases of aging brain.

## Introduction

Microglia are considered the tissue-resident macrophages that derive from the primitive macrophages produced in the yolk sack. These primitive cells migrate and reach the central nervous system (CNS) at early embryonic stage, prior to development of the bone marrow hematopoietic system (Ginhoux et al., 2010; Schulz et al., 2012; Yona et al., 2013), where they expand and colonize the entire CNS. Depending on the species, microglia account for 5–20% of the total glial cells present in the adult brain (Lawson et al., 1992). Secluded by the blood brain barrier (BBB) and evenly distributed through the brain parenchyma, they form an autonomous population distinct from the peripheral circulating monocytes or macrophages (Ginhoux et al., 2010; Schulz et al., 2012). Microglia play important roles in chronic neurodegeneration as well as in acute lesions in the brain including trauma and stroke, when the BBB is compromised. They express a wide range of receptors allowing them to respond to large number of cytokine signals from other cells circulating in blood and tissue. Therefore, until recently, microglia were mainly seen as the immune-competent cells of the CNS forming the first line of defense against invading pathogens or in case of injury or disease (Nimmerjahn et al., 2005). Recent literature, however, has demonstrated more sophisticated functions of these cells going beyond immune surveillance. Of particular importance, microglia actively participate in plasticity and maintenance of the adult CNS by secreting cytokines and neurotrophic factors including BDNF (Parkhurst et al., 2013) and refining the neuronal circuit by pruning synapses and axonal terminals (Tremblay and Majewska, 2011; Parkhurst et al., 2013; Salter and Beggs, 2014). Hence, in addition to immune surveillance and response, microglia have a number of additional distinct functions compared to immune cells in the blood. Moreover, while monocytes readily replenish from the bone-marrow hematopoietic stem cells, microglia have ~20–30 folds slower self-renewing capacity compared to them under homeostatic conditions (Elmore et al., 2014). Because life span of CNS microglia is longer, they are more prone to accumulating aging-related changes (Gehrmann and Banati, 1995). In addition, it has been proposed that a subtype of monocytes (Ly-6C^hi^CCR2) could replace microglia by being recruited from the blood circulation and sub-sequentially differentiated into microglia (Mildner et al., 2007; Varvel et al., 2012). However, to which extend these cells could take over different microglia functions is still not yet fully understood.

Hence, the view that microglia act as simple CNS scavengers, cleaning debris and dead cells, is out of date. The microglia are dynamic cells with the capacity of broad spectra of supportive as well as destructive functions in health and disease. The balance between these two opposing roles—undermined by infections, trauma or stroke challenges—are critical for the course of neurodegenerative diseases. Microglia have a high level of plasticity allowing them to change their shape and function in response to environmental cues (Saijo and Glass, 2011). After injury or over time with the aging, their morphology is progressively altered. For example, abnormal microglia morphology and dysfunction have been linked to many neurodegenerative diseases and psychiatric disorders including Alzheimer’s disease (AD), Parkinson’s disease (PD) and Rett syndrome (Prinz and Priller, 2014). While microglia morphology in general is a good indication of their functions, it is important to assess their cytokine profiles and interactions with the surrounding cells to determine their exact roles in a given situation.

Here we would like to argue that microglia’s function and morphology considerably change with the aging. Thus, their response to acute CNS lesions (stroke or trauma) depends on the age of the insult. On the other hand, any acute lesion could confer additional imprints to microglia function, thereby weaken their protective response to future insults and accelerate the aging of the brain.

## Microglia in the young healthy brain

While microglia have mainly been studied in the context of disease, recent studies yielded important insights on their significance also in the healthy brain specifically on their contribution to the maintenance of brain’s homeostasis (for review, see Schafer et al., 2012, 2013; Wu et al., 2013; Salter and Beggs, 2014). Microglia monitor changes in their environment with their long and motile processes, an activity that is facilitated by their positioning in a grid like fashion within the brain parenchyma. Because of their motility and positioning, they could scan the entire brain tissue every few hours (Davalos et al., 2005; Nimmerjahn et al., 2005). Such microglia dynamics are age-dependent and seem to slow down with the aging (Hefendehl et al., 2014). The homeostatic role of microglia has been linked at least in part to their phagocytic activity to sculpt the developing and young adult brain. Microglia contribute to elimination of sub-numeral Purkinje neurons in the developing cerebellum, a process potentially triggered by free radical release from the microglia (Marín-Teva et al., 2004). However, the molecular mechanisms triggering the engulfment of neurons by microglia are poorly uncovered. One idea is that microglia may recognize the apoptotic targets cells via a “receptor-ligand” interaction as it has been reported during the neurogenesis in the adult hippocampus. The subgranular zone (SGZ) of the dentate gyrus gives rise to numerous new cells. Only a small subset of these cells can reach to the maturity of a neuron and integrate into the hippocampal circuitry while most of them die. During these events, microglia rapidly dispose the dead cells and clear the neurogenic compartment long before migration of the remaining cells to the granular layer (Sierra et al., 2010).

Microglial might also be monitoring neuronal activity via transient contacts with dendritic spines and synapses (Wake et al., 2009; Tremblay et al., 2010). When needed, microglia may prune these dendritic spines and synapses via phagocytic mechanisms (Davalos et al., 2005; Nimmerjahn et al., 2005). For example, physical elimination of the contacted synapse by microglia occurs after an episode of light deprivation in the visual cortex or in the penumbra upon cerebral ischemia (Wake et al., 2009; Tremblay et al., 2010). Proposed microglial phagocytosis of neurons, dendritic spines and axonal shafts depends on the “eat me” and “don’t eat me” signals exposed at the neuronal surface (Brown and Neher, 2014). Local flipping of the plasma membrane phospholipids exposing phosphatidylserines to the external layer and synapse tagging with the complement C3 or C1q proteins are part of the signals mediating phagocytosis (Stevens et al., 2007; Berg et al., 2012). Conversely, neurons expressing CD47 and sialylated glycoproteins inhibit this process by binding to the microglial receptors signal regulatory protein 1α (SIRP1α) and sialic acid-binding immunoglobulin-like lectins (SIGLECs), respectively (Brown and Neher, 2014).

## Microglia in aging brain

Microglia morphology, number and dynamics are altered throughout the aging (Harry, 2013). Studies in young vs. aged retina (Damani et al., 2011) or brain (Hefendehl et al., 2014) have revealed that microglia exhibit age-related soma volume increase, shortening of their processes and loss of homogeneous tissue distribution. In addition, microglia exhibit slower acute and sustained chronic post-injury response, reminiscent of a prolonged inflammatory response (Damani et al., 2011; Hefendehl et al., 2014). Microglia can display swellings, varicosities and retraction of the ramifications, which are indication of unhealthy microglia (Mrak and Griffin, 2005; Miller and Streit, 2007; Norden and Godbout, 2013). Aging *per se* can reduce microglia phagocytic capacities for endogenous proteins such as Abeta peptides (Floden and Combs, 2011; Harry, 2013) and reduce their expression of phagocytosis and/or endocytosis genes (Orre et al., 2014). In addition, *in vitro* data indicate that microglia in the aged brain express more MHC-II molecules and become less sensitive to regulatory signals, such as transforming growth factor beta 1 (TGF beta-1) or colony stimulating factor 1 (CSF1; Rozovsky et al., 1998). During their life span, episodes of systemic inflammation and cytokine stimulation can instruct microglia and increase their reactivity. This mechanism of exposure to multiple noxious stimuli is called priming (Perry and Holmes, 2014). Along with the priming, accumulation of mutations and DNA damage with the aging (Mrak and Griffin, 2005), can lead microglia to gradually acquire resistance to regulation (Norden and Godbout, 2013; Perry and Holmes, 2014).

Upon activation, microglia density is increased several folds (Erturk et al., 2012), which eventually drops back to normal levels during the recovery phase (Streit, 2006). This reduction of microglia numbers in a pathological context is reestablished by apoptosis through activation-induced cell death (AICD), a mechanism triggered by interferon gamma (Takeuchi et al., 2006). Moreover, accumulation of functional and morphological alterations over time also implies that microglia could die independently of AICD, as shown in human brain (Streit, 2004; Streit and Xue, 2009). Potentially these mechanisms could lead to a substantial decrease in the number of microglia, because the proliferation rate is quite low in physiological conditions. While the number of mitotic divisions achieved before death is not known (Saijo and Glass, 2011), telomere shortening along with a significant decrease of telomerase activity—a marker of aging and senescence—in microglia have been reported during normal aging (Flanary et al., 2007). Taken together, this suggests that aged microglia decline in homeostatic functions and become susceptible to deterioration.

Parabiosis experiments have revealed that the source of microglia replenishment depends on the BBB integrity (Wright et al., 2001; Ajami et al., 2007). When the BBB is compromised, Ly-6C^hi^CCR2^+^ monocytes are recruited from the blood circulation (Mildner et al., 2007). Alternatively, when the BBB is intact, global depletion of microglia by blockage of CSF1 mobilizes a pool of latent progenitors, which, probably originate from the neuroectoderm—a different source than original microglia pool—as they express the specific marker Nestin (Elmore et al., 2014). Whether these substituting cells are really able to recapitulate the very different functions of microglia is unclear. It is possible that reactive microglia during aging could be deriving from the neuroectoderm lineage. Hence, future studies need to characterize different subtypes of microglia in the aging brain and their origins to determine which types support neuronal survival and which are detrimental to neuronal health.

## Microglia in brain lesions (stroke and trauma)

**Figure 1:**
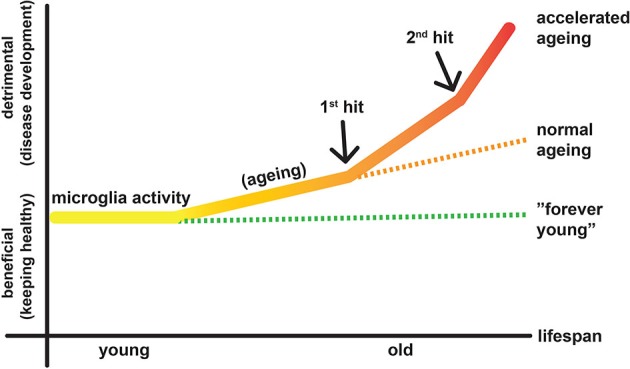
**Diagram illustrates putative activities of microglia in aging and lesioned brain**. Aging constitutes the continuous factor that transforms some of the microglia to the destructive mode, which may contribute to development of diseases. When there is a lesion, e.g., TBI or stroke (1st hit), some microglia become M1 type, which causes further neurodegeneration while a larger population is still M2 type, which helps healing the lesion environment. In addition, infiltration of the blood-derived immune cells (monocytes/macrophages, lymphocytes) forms the second wing of the inflammatory response and may contribute to neuronal protection and disease development.

After a brain lesion, e.g., induced by TBI or ischemic stroke, neuroinflammatory responses are prominent (Liesz et al., 2011). The acute stage begins with the local death of damaged neurons via necrosis and apoptosis (Raghupathi, 2004). It is associated with a rapid inflammatory response involving both resident microglia and infiltrating blood-borne immune cells (neutrophils, monocytes, leukocytes; for a detailed review please refer to Famakin, 2014). This initial neuroinflammation can be both destructive and beneficial depending on the subtype and spatiotemporal distribution of the inflammatory cells and the environmental cues surrounding them (Kreutzberg, 1996; Ramlackhansingh et al., 2011; Aguzzi et al., 2013; Jeong et al., 2013). Neurodegeneration progresses long after acute lesion, perhaps throughout the remaining lifetime, which may result in chronic neurological complications such as dementia (Smith et al., 1997; Pierce et al., 1998; Bramlett and Dietrich, 2002). However, how the initial injury spreads to the rest of the brain and how microglia is involved in this chronic neurodegeneration process are currently unknown (Masel and DeWitt, 2010). Human MRI and PET studies indicate that white matter track pathology after stroke contributes to a secondary degenerative process in the corresponding cortex (Duering et al., 2012) that seems to be associated with microglia/macrophage activation (Radlinska et al., 2009). Could a possible chronic neuroinflammation be a major contributor to long-term degeneration of the brain? In support of this hypothesis, GWAS studies demonstrate that inflammation-related TREM2 (Guerreiro et al., 2013; Jonsson et al., 2013) and CD33 (Hollingworth et al., 2011; Naj et al., 2011) genes are risk factors for AD. In addition, increased microglial response is associated with enhanced pathology and behavioral decline in an experimental model of dementia (Boimel et al., 2010). To our view, detrimental inflammatory response is exacerbated even by aging alone. Additional insults in the brain (e.g., acute injury) might catalyze this inflammatory response and further accelerate aging of the brain (Smith et al., 2013; Jacquin et al., 2014; Figure 1).

Determination of the exact role of microglia in the lesioned CNS is complicated due to the fact that resident microglia cannot easily be distinguished from the blood-borne infiltrating immune cells (e.g., macrophages/monocytes), which come through the leaky BBB. Hence, majority of studies in the context of injuries provided limited information on the specific role of microglia (Hellwig et al., 2013; Perego et al., 2013; Yamasaki et al., 2014). Yet, recent studies demonstrated that indeed microglia and blood-derived immune cells differ in their gene expression signatures, hence, possibly in their functions (Butovsky et al., 2014; Prinz and Priller, 2014). In line with these findings, studies from the traumatic spinal cord injury indicate that blood-derived infiltrating macrophages, but not the resident microglia, are responsible for the secondary axonal dieback (few weeks after the initial insult) (Evans et al., 2014). Similarly, there is supporting data that blood-derived macrophages initiate demyelination in the EAE model, while microglia cleanup the debris and provide trophic support to maintain the tissue homeostasis during the early phases of the disease (Yamasaki et al., 2014). Hence, it is reasonable to consider that the short-lived blood-derived macrophages/monocytes and the long-lived (Elmore et al., 2014) resident microglia are different cell populations with only partially overlapping functions. In the future, accumulation of knowledge on the specificity of each immune cell type (e.g., via the recently generated CCR2-RFP/CX3CR1-GFP transgenic mouse (Saederup et al., 2010)) will be critical to tackle CNS diseases by targeting only the destructive immune cells while preserving the beneficial ones.

Microglia express a repertoire of various receptors such as TREM2, FcγRs, MHC-II, CD200R, RAGE, CX3CR1 (fractalkaline), CXCR3 and 4, purinergic receptors, Toll-like receptors 2 and 4, galectins 1 and 3, scavenger receptors (e.g., CD36), CD47, integrins and SIRPα (Hu et al., 2014). Thereby, they provide both pro-inflammatory and anti-inflammatory response, in a varying range depending on the signals dictated by their environment (Hu et al., 2014, 2015; Peferoen et al., 2015). In the normal brain, it is now understood that microglia activity is repressed by their repeated contacts with normal neurons via inhibitory inputs such as CD200, CX3CL1, CD47, CD22, CD172 or TREM2 (Hellwig et al., 2013). Under acute neuronal injury, inhibitory signals are reduced and danger stimuli (danger-associated molecular patterns, DAMPs) are released (Weinstein et al., 2010). These modifications trigger changes in the microglial response to the environment, collectively resulting in microglia activation, proliferation, migration and response (Patel et al., 2013). The type of microglia response can also vary depending on the mechanisms triggering the lesion (Cherry et al., 2014) (e.g., non-autoimmune, pathogen-associated triggered inflammation vs. adaptive immunity) (Zindler and Zipp, 2010).

Thus, on one hand, microglia can encapsulate dangerous foci and remove the cellular debris via phagocytosis in order to protect the surrounding CNS tissue; on the other hand, they can harm the injured CNS via propagation of inflammation, pro-inflammatory cytokine secretion, antigen-presentation (MHC-II positive) and further immune cell recruitment. Eventually microglia get “deactivated” or cleaned-up by adjacent cells via poorly understood processes that are guided by local and systemic homeostatic signals (Hristova et al., 2010; Saijo and Glass, 2011; Starossom et al., 2012; Patel et al., 2013).

**Figure 2:**
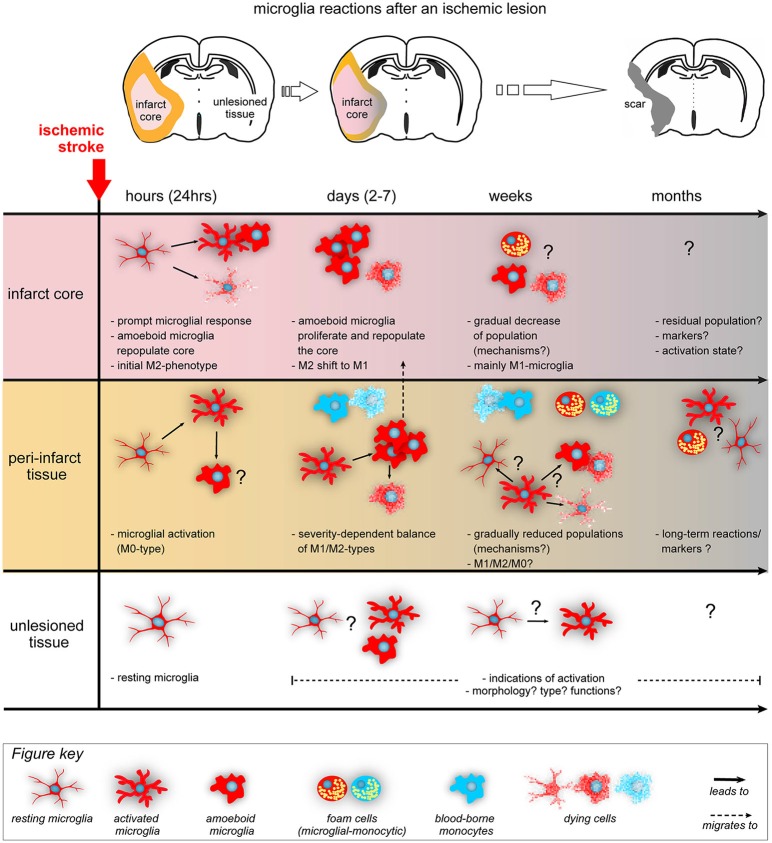
**Acute lesions trigger morphological and functional changes from resident microglia**. The diagram summarizes the main microglia’s temporal (hours to months) and spatial (infarct core, peri-infarct area and unlesioned tissue) kinetics after an ischemic lesion (Ito et al., 2007; Perego et al., 2011, 2013; Hu et al., 2012; Morrison and Filosa, 2013; Patel et al., 2013; Taylor and Sansing, 2013). Infarct core (pink, upper panel) is surrounded by penumbra (orange, middle panel) in the acute phase, a peri-infarct region in the intermediate phases and turns into a scar (gray, with or without cavitation depending on the species) in the chronic phase. During acute phase (first 24 **h**), microglia are the first to respond to the lesion: unless they die immediately by the ischemic processes of the core, they are activated gaining an M2 functional polarization. In the peri-infarct region, microglia are activated but are initially not polarized (M0). In the following **days**, microglia are further activated in the peri-infarct area, proliferate, migrate to the core to repopulate the corresponding cells and some of them die. Depending on the ischemic severity and neuronal damage in the peri-infarct regions, microglia gradually acquire different, region-dependent, polarization states and eventually shift from M2 to M1 microglia as core expands to penumbra and neurons die. At this period, blood-borne monocytes (blue cells) and lymphocytes and neutrophils (not shown here) infiltrate mainly the peri-infarct regions. During the subchronic phase (**weeks**), the core is further cleared from debris (amoeboid microglia turn into foam cells or die) and microglia in the peri-infarct area possibly follow regionally different paths (resting, activation or death), under processes not well studied so far. Foam cells are present (coming from both resident and blood-macrophages), while the numbers of blood-borne cells gradually decline. In the chronic phase (**months**), there are indications of long-term microglial activation and presence of residual foam cells in the peri-infarct tissue, with unknown significance so far. Importantly enough, the **unlesioned tissue** is not well studied so far but probably holds populations of activated microglia that respond or facilitate local degenerative processes. For the simplicity of the figure, we have not included the secreted cytokines produced by the microglia, their changes in their receptors and the contribution of other immune cells. M0: non-polarized microglia, M1: pro-inflammatory or classically activated microglia, M2: anti-inflammatory or alternatively activated microglia (Patel et al., 2013), “?” indicate lack of detailed information.

Stroke and TBI initiates a cascade of events (Iadecola and Anrather, 2011) that includes all cellular components of the brain as well as a systemic response from the periphery (Lee et al., 2014). We know that in both ischemic stroke and TBI (Nimmerjahn et al., 2005), microglia respond quickly within the first minutes-hours after the insult (Gelderblom et al., 2009) and the overall neuroinflammatory milieu seems to a peak at around day 5 post-lesion (Turtzo et al., 2014). Interestingly, the initial microglial response in stroke seems to be primarily helping the tissue repair (Hu et al., 2012; Figure 2). These microglia secrete and balance anti-inflammatory cytokines and growth factors (IGF1, TGFb1, neurotrophic factors) to promote tissue repair (Wang et al., 2013), indicating that their primary effect after sub-acute ischemia or TBI is to protect the brain and not to kill it (Patel et al., 2013). As mentioned in the review by Hellwig et al. (2013), it is unlikely that the real reason for the presence of numerous inflammatory cells in the vulnerable brain is just to cause harm. In line with this assumption, it has been shown that the enhancement of the microglial population by transplantation of microglia can ameliorate the ischemia-induced injuries via multiple mechanisms, such as upregulation of neurotrophic factors or an active interaction and engulfment of the few neutrophils that might migrate perivascularly after stroke (Neumann et al., 2006, 2008, 2015; Narantuya et al., 2010; Perez-de-Puig et al., 2015). However, microglia dynamically change their phenotypes and they react to the ongoing neuronal death in the peri-infarct regions (Hu et al., 2012) as the lesion extends from core to penumbra over time (Lee et al., 2014; Figure 2). Such a change is dictated by the dynamic local ischemic cues (cytokines, chemokines, cells, complement molecules, DAMPs) (Hu et al., 2014). In the lesioned brain, debris are removed via phagocytosis largely by microglia and a lesser extent by infiltrating macrophages (Fu et al., 2014). Removal of debris is beneficial for the tissue and its regeneration (Neumann et al., 2009) but large amounts of debris can overload the microglia and render them dysfunctional over time (Li, 2013). Such a dysfunction can lead to tissue aging as we discuss below.

It is now more evident that neuroinflammation can affect neuronal degeneration and recovery depending on the age of the organism at the time of insult. In other words, we should consider microglia as the brain’s guardian of the innate immune compartment that responds to danger and shape a reaction (beneficial or not) (Kigerl et al., 2014) based on their history. Aged microglia are more sensitive to inflammatory stimuli and become resistant to regulation by exposure to multiple noxious stimuli during the life-span of the organism (Norden and Godbout, 2013; Perry and Holmes, 2014). Aging *per se*, can imbalance the repertoire of receptors docked at the membrane and thereby alter the microglial response to environmental cues. Aging decreases some silencing receptors on microglia, e.g., CX3CR1 (Wynne et al., 2010) and CD200 (Lyons et al., 2007), while increases some of the activating ones, thereby priming microglia to become more readily activated upon any trigger (Wong, 2013; Raj et al., 2014). Aged microglia seems to have higher proliferative capacity in response to injury compared to younger adult animals, for example, as observed in facial nerve crush injury (Miller and Streit, 2007) or in mild ischemic injuries (Yan et al., 2014). Moreover, in an aged organism, where a chronic and subtle infection could reside, the intrinsic state of microglia is also *per se* different (increased proinflammatory response) (Püntener et al., 2012) and such microglia may have maladjusted protective response in case of an acute insult. Increased pro-inflammatory or reduced cyto-protective responses related to aging of the organism are indeed a common feature of long-lived resident macrophages reported in various organs including liver (Okaya et al., 2005; Bouchlaka et al., 2013). Conversely, repeated acute lesions can exhaust microglia and reduce their phagocytic function, resulting in chronic, unresolved, sterile inflammation that may propagate over months/years (Li, 2013). The most striking examples for the necessity of a healthy phagocytosis by microglia come from AD (Njie et al., 2012) and multiple sclerosis (Napoli and Neumann, 2010) studies, in which microglial phagocytosis—that is necessary for the clearance of aggregates (e.g., Abeta) or debris—is reduced (Floden and Combs, 2011). In addition to being exhausted by workload, autophagy dysfunction and mitochondrial DNA damage seen in microglia could further contribute to the brain aging and development of neurodegenerative diseases (Nakanishi and Wu, 2009). Eventually, since microglia continuously shape neuronal circuitry and their functions are altered in the post-lesioned brain (Wake et al., 2009), they could also participate in defective circuit remodeling removing not only the degenerating non-functional synapses but also eliminating healthy synapses. Overall, we speculate that multiple acute lesions over the lifespan accelerate aging of the CNS by priming microglia bit-by-bit until they lose their homeostatic and/or repairing capacity (Figure 2).

## Conclusion

Microglia identity is progressively altered in the aging brain leading to both immunological and homeostasis dysfunctions. In addition to age related decline, microglia accumulate alterations rendering them weaker against protection of the brain after an ischemic or traumatic insult. On the other hand, lesions can prime microglia to age faster, which in return can certainly contribute to escalation of neurodegenerative diseases (Figure 1). In fact, resident microglia, which can be imprinted by multiple exposures to insults in the aging brain, should be regarded as “veteran” cells. Therefore, investigating the molecular and cellular mechanisms underlying long-term changes in microglia’s identity in response to acute injuries at different times would provide valuable insight for better understanding the aging progression. We believe that novel strategies aiming to reverse the microglia aging could carry high therapeutic potentials for both acute injuries and neurodegenerative diseases.

## Conflict of interest statement

The authors declare that the research was conducted in the absence of any commercial or financial relationships that could be construed as a potential conflict of interest.
